# Supplementary material for: The L-Type Lectin-like Receptor Kinase Gene TaLecRK-IV.1 Regulates the Plant Height in Wheat
Source: Int J Mol Sci. 2022 Jul 26;23(15):8208. doi: 10.3390/ijms23158208 (PMC9332437; doi:10.3390/ijms23158208)
Supplement: Supplementary file 1 [file ijms-23-08208-s001.zip › ijms-1738495-supplementary.pdf]

Supplemental Table S1. Primers and their sequences in this study

marker	Sequence (5′-3′)	Usage	Annealing temperature (°C)
[a]*TaLecRK-Q-F*	GCATTCGGTGTGTTCCTCC	Quantitative RT-PCR	56
[a]*TaLecRK-Q-R*	AACCTCCTGTGTGTCAAACTTTT	Quantitative RT-PCR	56
TaLecRK-V-F	CAGGCTAGCCTAGATCGTGGGAAGTCGGT	Cloning gene fragment for VIGS	55
TaLecRK-V-R	AATGCTAGCTCTTCTAAATGAAACATCAAAAAT	Cloning gene fragment for VIGS	55
TaLecRK-FL-F	ATACTACTGAGTTTTCTTTTGTCC	Cloning full length sequence	57
TaLecRK-FL-R	TAGGGCTGTGATTCTGAGGC	Cloning full length sequence	57
TaLecRK-CS-F	GCATTCGGTGTGTTCCTCC	Chromosomal location	56
TaLecRK-CS-R	AACCTCCTGTGTGTCAAACTTTT	Chromosomal location	56
[b]7A homolog gene – Q-F	TCAAGGCAAGCAATGTTCTCT	Quantitative RT-PCR	56
[b]7A homolog gene – Q-R	CACAAGCTCTGGTGCAAGGTA	Quantitative RT-PCR	56
7A- V-F	CAGGCTAGCCTCAAGCTTAACAGCCGCA	Cloning gene segment for VIGS	57
7A- V-R	AATGCTAGCCGCCATGAGAGTGGAGAGG	Cloning gene segment for VIGS	57
7A- FL-F	CTGCTCATACTCCTGCTGT	Cloning full length	57
7A- FL-R	TCTTCTAAATGAAACATCAAAAAT	Cloning full length	57
[c]*TaGA20ox-Q-F*	GGCGACACCTTCATGGCGC	Quantitative RT-PCR	56
[c]*TaGA20ox-Q-R*	ACCACCTTGTCCATCTCCGG	Quantitative RT-PCR	56
[d]*TaGA3ox-Q-F*	AGGTCGCCGCCGTCGAGTCC	Quantitative RT-PCR	56
[d]*TaGA3ox-Q-R*	GATATCCGGTCGCTGTCC	Quantitative RT-PCR	56
TaActin-Q-F	CACTGGAATGGTCAAGGCTG	Quantitative RT-PCR	56
BSMV-CP-F	TGACTGCTAAGGGTGGAGGA	PCR	56
BSMV-CP-R	CGGTTGAACATCACGAAGAGT	PCR	56

Note: The underlined sequences indicate the restriction enzyme site. Primer efficiencies: a = 108%, b = 107%, c = 101%, d = 103%

Supplemental Table S2. Percentage of the amino-acid identity between the deduced *TaLecRK-IV.1* protein and other protein sequences from the database

Species/sub-species	Protein name	Database accession number	Percentage of the amino-acid identity with the deduced protein sequence of *TaLecRK-IV.1*
Triticum aestivum	Lectin receptor kinase 2	ACL36476.1	93.49%
Aegilops tauschii	L-type lectin-domain containing receptor kinase IV.1-like	XP_020162166.1	93.07%
Setaria italica	L-type lectin-domain containing receptor kinase IV.1	XP_022680875.1	81.22%
Panicum hallii	L-type lectin-domain containing receptor kinase IV.1-like isoform X1	XP_025798945.1	82.86%
Oryza brachyantha	L-type lectin-domain containing receptor kinase IV.1-like (predicted)	XP_00666406.1	78.39%
Oryza sativa	L-type lectin-domain containing receptor kinase IV.1	XP_015645135.1	73.80%
Dichanthelium oligosanthes	L-type lectin-domain containing receptor kinase IV.2	OEL23611.1	73.57%
Zea mays	L-type lectin-domain containing receptor kinase IV.1	XP_008653275.1	71.17%
Brachypodium distachyon	L-type lectin-domain containing receptor kinase IV.1	XP_003580202.1	64.68%
Sorghum bicolor	L-type lectin-domain containing receptor kinase IV.1	XP_002446842.1	64.67%
Elaeis guineensis	L-type lectin-domain containing receptor kinase IV.1-like	XP_010943349.1	62.67%
Musa acuminate ssp. malaccensis	L-type lectin-domain containing receptor kinase IV.1-like (predicted)	XP_018673595.1	61.41%
